# Electroconvulsive therapy-induced mania: a case report

**DOI:** 10.1186/1752-1947-3-94

**Published:** 2009-11-02

**Authors:** Omer Saatcioglu, Mehmet Guduk

**Affiliations:** 1 Bakirkoy Research and Training Hospital for Psychiatry, Neurology and Neurosurgery, Istanbul, Turkey

## Abstract

**Introduction:**

Despite its controversial history, electroconvulsive therapy is generally an effective treatment with few serious side effects. One rare but troublesome side effect of electroconvulsive therapy is mania.

**Case presentation:**

A 33-year-old Turkish woman developed mania on three separate occasions after receiving electroconvulsive therapy for severe depressive episodes.

**Conclusion:**

Patients who experience electroconvulsive therapy-related mania should be evaluated for alternative treatments when presenting with severe depression.

## Introduction

Electroconvulsive therapy (ECT) has been in use for almost half a century. It is a safe and efficient treatment method for numerous psychiatric disorders if scientific criteria and principles are observed in both the selection of patients and the implementation of the treatment [1-3]. ECT is known to cause certain side effects including cognitive dysfunction, cardiovascular problems, and in rare cases, mania [4,5]. In ECT-related mania, one may choose either to cease or to continue treatment [2,6,7].

## Case presentation

A 33-year-old Turkish woman presented with a decrease in psychomotor activation (PMA) and self-care, dysphoria, insomnia, and persecutory delusions. She was hospitalized with the preliminary diagnosis of "major depression with psychotic features," according to DSM-IV criteria. The patient was not addicted to alcohol or any illicit substances. Her score on the 17-item Hamilton Depression Rating Scale (HAMD-17) was 46, indicating very severe depression. Full laboratory investigations were in the normal range. She was commenced on haloperidol, 20 mg/day, and biperiden, 4 mg/day, and was given seven sessions of ECT, as described in detail below. Increases in PMA, euphoria and grandiosity were observed after the seventh ECT treatment. Subsequently ECT was stopped, and lithium, 900 mg/day, was added to the treatment. Her score on the Young Mania Rating Scale (YMRS) was 28, and her score on the HAMD-17 was less than 7. Her mania and depression severity were, respectively, moderate and normal. The patient was discharged with a prescribed treatment of lithium, 900 mg/day, (serum level: 0.6 mg/dL) and chlorpromazine, 300 mg/day.

Although she complied fully with her medications initially, she stopped taking them 2 years later during pregnancy. Following her pregnancy, at the early phase of the postpartum period, she was hospitalized again with a diagnosis of "major depressive episode." Her HAMD-17 score of 42 indicated very severe depression. Initially, the patient was commenced on diazepam, 15 mg/day, which was stopped and followed by ECT. She was observed to be too active after the second ECT, and it was stopped after the third ECT session. Symptoms at this point included excessive speaking, euphoria and restlessness. Her YMRS score of 24 indicated moderate severity of mania. Since this was judged to be an ECT-induced mania, she was commenced initially on haloperidol, 20 mg/day, and biperiden, 4 mg/day, which was changed to lithium, 900 mg/day. She was discharged with a recommended treatment of lithium, 1200 mg/day (serum level: 0.76 mg/dL), and chlorpromazine, 300 mg/day.

After 21 months, she was re-hospitalized with the initial diagnosis of "major depressive episode". She presented with a depressive melancholic mood, and suffered from persecutory delusions. Her HAMD-17 score was 42. Laboratory investigations were in the normal range except thyroid function test (TFT) levels. There was a decrease in fT4 0.8 ng/dl (normal range: 0.93 to 1.7 ng/dl) level, and a normal range in fT3 3.48 pg/ml (normal range: 2.0 to 4.4 pg/ml) and TSH 2.03 uIU/ml (normal range: 0.27 to 4.2 uIU/ml) levels. After four weeks, her TFT levels were re-measured in the normal range. Also in her previous episodes, TFT levels were found to be normal. Treatment with haloperidol, 30 mg/day, biperiden, 4 mg/day, and diazepam, 10 mg/day, was not satisfactory. The patient had total of five ECTs, but was discharged from the hospital against medical advice, which was then followed by some improvement.

Six months later, she was re-hospitalized with a similar presentation, and was commenced on haloperidol, 30 mg/day, biperiden and 4 mg/day. After five sessions, ECT was stopped due to the emergence of symptoms of excessive speaking, lack of calmness, elation and restlessness. Her YMRS score was 24. Laboratory investigations were in the normal range. She was discharged from the hospital with a medication consisting of haloperidol, 20 mg/day, biperiden, 4 mg/day, and carbamazepine, 400 mg/day. This treatment led to a partial improvement in her major psychiatric symptoms. The patient attended the outpatient clinic in the month after her discharge from hospital. She had full remission, which was taken to imply an improvement in both her clinical symptoms and functional disability.

The patient was given unmodified ECT with no anaesthesia during three hospitalizations, and modified ECT with anaesthesia and muscle relaxants during the last hospitalization. In this case, ECT was administered in three sessions a week for all inpatient treatments. ECT was recommended, and consent was obtained from the patient and her husband. This informed consent procedure was applied according to approved legal and ethical practices for people with mental illness in Turkey. A Thymatron^® ^system IV-Integrated ECT Instrument (Somatics, LLC; Lake Bluff, IL) was used. Standard bifrontotemporal electrode placements were employed for bilateral ECT. In the first ECT session, a stimulus dose was selected to produce an intense seizure. For bilateral electrode placements a dose in miliCoulombs (mC) equal to 3.5 to 4 times the patient's age sufficed (an initial dose of 126 mC, with an ECT dose ranging from 118 mC to 120 mC). A seizure threshold of 126 mC resulted in a 129-second EEG, a 32-second motor seizure, and a postictal suppression index (PSI) of 80%. For other ECTs, the stimulus dosage range was between 118 mC to 120 mC, which elicited a seizure duration of 15 to 26 seconds. Ventilation was applied using a face mask during seizures, and oxygenation of the patient was monitored.

In summary, the patient was hospitalized on four separate occasions with the diagnosis of "major depression with psychotic features," and developed ECT-related mania during three of these episodes. All depressive episodes were attributable to a failure to use medication regularly or to a cessation of medication. Eventual clinical improvement in all episodes was achieved by resumption of psychiatric medication.

## Discussion

Electroconvulsive therapy is generally used on severely depressed patients when other forms of therapy, such as medications or psychotherapy, have not been effective, or cannot be tolerated, or, in life-threatening cases, will not help the patient quickly enough. ECT also helps patients who suffer with prolonged or severe episodes of mania, although mood stabilizers and antipsychotics are the mainstay of mania treatment [2,5,7].

Researchers still do not know how ECT works. There are several major theories that attempt to explain why it works. The neurotransmitter theory suggests that ECT works like anti-depressant medication in changing the way receptors receive mood related chemicals like serotonin [8-10]. The anticonvulsant theory proposes that the induced seizures teach the brain to resist seizures. This effort to inhibit seizures dampens abnormally active brain circuits, stabilizing mood [8-10]. The neuroendocrine theory hypothesizes that the seizure causes the hypothalamus to release chemicals that cause changes throughout the body [9].

ECT affects the brain by increasing metabolism and blood flow to certain parts of the brain; however, it is not known how this increased blood flow alleviates depression [10]. Recent studies in animals suggest that ECT has potent effects in bolstering neuronal survival. In common with chemical antidepressant treatments, CT enhances the expression of a neuroprotective protein, brain-derived neurotrophic factor (BDNF), which antagonises the neurotoxic effects of stress on the brain [8].

Angst's results show that 64 of the 908 patients (7.0%) admitted for depression switched to hypomania or mania in a study between 1920 and1982. The switch rate is mainly explained by polarity; patients with a previous history of mania and/or hypomania have switch rates of 21% (mania to depression) to 29% (depression to mania) [11,12].

While many switches observed in depressive patients may be due to disease course, we believe that the timing in this case is highly suggestive of ECT-related mania. Interestingly, our patient never experienced mania without first undergoing ECT. Some clinicians argue that in cases such as ours, limbic stimulation by ECT exceeded the affective target, resulting in mania [13]. Once the patient became manic, we had the choice to continue or to cease ECT. Because the severity of mania was moderate, the decision was made to use a pharmacologic approach [14-16]. Medications proved adequate in treating a future episode of major depression with psychosis.

## Conclusion

In deciding whether to administer ECT to a patient who has experienced ECT-related mania, one must weigh the risks and benefits of such a treatment. The severity of our patient's depression seemed to indicate ECT on several admissions, while the side effect of mania was a substantial risk. We suggest that a history of ECT-related mania should be considered when choosing treatments in patients with depressive episodes.

## Abbreviations

ECT: Electroconvulsive therapy; HAMD-17: 17-item Hamilton Depression Rating Scale; YMRS: Young Mania Rating Scale; PMA: psychomotor activation; TFT: thyroid function test; mC: miliCoulombs; BDNF: brain-derived neurotrophic factor.

## Consent

Written informed consent was obtained from the patient for publication of this case report. A copy of the written consent is available for review by the Editor-in-Chief of this journal.

## Competing interests

The authors declare that they have no competing interests.

## Authors' contributions

MG was in charge of the overall care of the patient and OS involved in follow up care. OS researched the literature and prepared the manuscript with critical review from MG. Both authors read and approved the final manuscript.
